# Efficacy and Safety of Colchicine in Post–acute Myocardial Infarction Patients: A Systematic Review and Meta-Analysis of Randomized Controlled Trials

**DOI:** 10.3389/fcvm.2021.676771

**Published:** 2021-06-08

**Authors:** Carlos Diaz-Arocutipa, Jerry K. Benites-Meza, Diego Chambergo-Michilot, Joshuan J. Barboza, Vinay Pasupuleti, Héctor Bueno, Antonia Sambola, Adrian V. Hernandez

**Affiliations:** ^1^Vicerrectorado de Investigación, Universidad San Ignacio de Loyola, Lima, Peru; ^2^Programa de Atención Domiciliaria – EsSalud, Lima, Peru; ^3^Tau Relaped Group, Trujillo, Peru; ^4^Facultad de Medicina, Universidad Nacional de Trujillo, Trujillo, Peru; ^5^Facultad de Ciencias de la Salud, Universidad Científica del Sur, Lima, Peru; ^6^MedErgy HealthGroup, Inc., Yardley, PA, United States; ^7^Centro Nacional de Investigaciones Cardiovasculares, Madrid, Spain; ^8^Cardiology Department, Hospital Universitario 12 de Octubre, Instituto de Investigación Sanitaria Hospital 12 de Octubre (imas12), Madrid, Spain; ^9^Facultad de Medicina, Universidad Complutense de Madrid, Madrid, Spain; ^10^Centro de Investigación Biomédica en Red Enfermedades Cardiovasculares, Madrid, Spain; ^11^Department of Cardiology, University Hospital Vall d'hebron, Universitat Autònoma, Barcelona, Spain; ^12^Health Outcomes, Policy, and Evidence Synthesis Group, University of Connecticut School of Pharmacy, Storrs, CT, United States

**Keywords:** colchicine, myocardial infarction, atherosclerosis, inflammation, meta-analysis

## Abstract

**Background:** Inflammation plays a key role in atherosclerotic plaque destabilization and adverse cardiac remodeling. Recent evidence has shown a promising role of colchicine in patients with coronary artery disease. We evaluated the efficacy and safety of colchicine in post–acute myocardial infarction (MI) patients.

**Methods:** We searched five electronic databases from inception to January 18, 2021, for randomized controlled trials (RCTs) evaluating colchicine in post–acute MI patients. Primary outcomes were cardiovascular mortality and recurrent MI. Secondary outcomes were all-cause mortality, stroke, urgent coronary revascularization, levels of follow-up high-sensitivity C-reactive protein (hs-CRP), and drug-related adverse events. All meta-analyses used inverse-variance random-effects models.

**Results:** Six RCTs involving 6,005 patients were included. Colchicine did not significantly reduce cardiovascular mortality [risk ratio (RR), 0.91; 95% confidence interval (95% CI), 0.52–1.61; *p* = 0.64], recurrent MI (RR, 0.87; 95% CI, 0.62–1.22; *p* = 0.28), all-cause mortality (RR, 1.06; 95% CI, 0.61–1.85; *p* = 0.78), stroke (RR, 0.28; 95% CI, 0.07–1.09; *p* = 0.05), urgent coronary revascularization (RR, 0.46; 95% CI, 0.02–8.89; *p* = 0.19), or decreased levels of follow-up hs-CRP (mean difference, −1.95 mg/L; 95% CI, −12.88 to 8.98; *p* = 0.61) compared to the control group. There was no increase in any adverse events (RR, 0.97; 95% CI, 0.89–1.07; *p* = 0.34) or gastrointestinal adverse events (RR, 2.49; 95% CI, 0.48–12.99; *p* = 0.20). Subgroup analyses by colchicine dose (0.5 vs. 1 mg/day), time of follow-up (<1 vs. ≥1 year), and treatment duration (≤30 vs. >30 days) showed no changes in the overall findings.

**Conclusion:** In post–acute MI patients, colchicine does not reduce cardiovascular or all-cause mortality, recurrent MI, or other cardiovascular outcomes. Also, colchicine did not increase drug-related adverse events.

## Introduction

Atherosclerotic coronary artery disease (CAD) remains a leading cause of global morbidity and mortality (1), despite progress in medical and invasive treatment. There is a current interest in inflammation as a therapeutic target in patients with acute coronary syndrome (ACS) as it plays a central role in the destabilization of atherosclerotic plaques and ventricular remodeling after ACS (2). In recent years, some drugs with anti-inflammatory effects have been assessed in patients with CAD. Canakinumab, a monoclonal antibody that inhibits interleukin 1β (IL-1β), significantly reduced major cardiovascular events and high-sensitivity C-reactive protein (hs-CRP) levels in patients with a history of myocardial infarction (MI) and hs-CRP > 2 mg/L (3). However, canakinumab was associated with a higher incidence of fatal infections compared to placebo. In contrast, methotrexate did not show a significant reduction of cardiovascular events or hs-CRP levels in patients with a previous CAD (4). Given these discordant results, it was necessary to search for new anti-inflammatory drugs with an adequate safety profile.

Colchicine is a low-cost drug with wider anti-inflammatory properties that has been used for more than 200 years for the treatment of gout attacks and currently in other inflammatory diseases such as acute pericarditis and familial Mediterranean fever. Few systematic reviews have examined the effect of colchicine in patients with CAD (5–11), but none focused on post–acute MI patients. The analysis of its effect specifically on MI patients is important because of the intense inflammatory response associated with acute MI and the high risk for poor outcomes in these patients in whom the anti-inflammatory effect of colchicine may be more relevant. We conducted a systematic review and meta-analysis to assess the efficacy and safety of colchicine in post–acute MI patients.

## Methods

This review was reported according to the 2009 PRISMA (Preferred Reporting Items for Systematic Reviews and Meta-Analyses) statement (12) and was registered in the PROSPERO database (CRD42020177536).

### Search Strategy

We searched the following bibliographic databases: PubMed, EMBASE, Scopus, Web of Science, and Cochrane Central Register of Controlled Trials. Searches were conducted from inception to April 4, 2020, with an update on January 18, 2021. The search strategy for PubMed was adapted for use in the other databases (Supplementary Table 1). We applied the Cochrane highly sensitive search strategy for identifying randomized controlled trials (RCTs) in PubMed (13). There were no restrictions on language or publication date. Also, we conducted a hand search of reference lists of included studies and relevant review articles to further identify eligible trials. Additionally, we searched trial registries www.clinicaltrials.gov/ and www.who.int/trialsearch/ for completed as well as ongoing RCTs.

### Eligibility Criteria

Study inclusion criteria were as follows: (i) RCTs, (ii) adult patients (≥18 years of age) post–acute MI defined according to study authors, (iii) any dose and duration of colchicine as experimental group, and (iv) placebo or standard treatment as a control group. We excluded observational studies, systematic reviews, narrative reviews, editorials, letters to the editor, and abstracts.

### Study Selection

Two review authors (CDA and JJB) downloaded all titles and abstracts from electronic search to EndNote X8 software and duplicate records were removed. Titles and abstracts of the retrieved articles were independently screened by two authors (CDA and JBM) to identify potentially relevant studies. Two authors (CDA and JBM) independently screened the full text and registered reasons for the exclusion of studies. Any disagreement on title/abstract and full-text selection was resolved by consensus.

### Outcomes

Primary outcomes were cardiovascular mortality and recurrent MI. Secondary outcomes were all-cause mortality, stroke, urgent coronary revascularization, follow-up hs-CRP level, any adverse events, and gastrointestinal adverse events. We used author-reported definitions for all outcomes.

### Data Extraction

Information from each study was independently extracted by two authors (CDA and DCM) using a standardized data extraction form that was previously piloted. Any disagreement was resolved by consensus. If additional data were needed, we contacted the corresponding author through email to request further information. We extracted the following data: first author name, year of publication, country, type of RCT, sample size, population, age, sex, duration and dosage of colchicine, and primary and secondary outcomes.

### Risk-of-Bias Assessment

Two review authors (CDA and VP) independently assessed the risk of bias of each study using the Cochrane Risk of Bias 2.0 tool (13). This tool evaluates five domains: randomization process, deviations from intended interventions, missing outcome data, measurement of the outcome, and selection of the reported result. Overall, each RCT was judged as having a low, some concerns, or a high risk of bias. Any disagreement was resolved by a third author (AVH).

### GRADE Quality of Evidence

The Grading of Recommendations Assessment, Development, and Evaluation (GRADE) approach (14) was used to appraise the quality of evidence for all outcomes. In using GRADE methodology, we considered the following criteria to assess the quality of evidence: risk of bias, inconsistency, indirectness, imprecision, and publication bias. We generate the Summary of Findings table using the GRADEpro software.

### Statistical Analysis

All meta-analyses were conducted using the inverse-variance method and random-effects models with Hartung–Knapp adjustment given the small number of included trials (15). The between-study variance (τ^2^) was estimated using the Paule–Mandel estimator (16). We pooled risk ratios (RRs) and mean differences (MDs) with their 95% confidence intervals (95% CIs) for binary and continuous outcomes, respectively. Heterogeneity among studies was evaluated using the χ^2^ test (*p* < 0.10 was considered as the presence of heterogeneity) and the *I*^2^ statistic. Heterogeneity was defined as low if *I*^2^ <30%, moderate if *I*^2^ = 30–60%, and high if *I*^2^ > 60%. As the number of trials was <10, it was not possible to evaluate small-study effects (13). In the case studies that reported only median and interquartile range, the mean and standard deviation were estimated using the method by Wan et al. (17). A pre-specified subgroup analyses were conducted according to (i) colchicine dose (0.5 vs. 1 mg/day), (ii) time of follow-up (<1 vs. ≥1 year), and (iii) treatment duration (≤30 vs. >30 days). In sensitivity analyses, we assessed (i) all meta-analyses performed without the Hartung–Knapp adjustment and (ii) only studies with a low risk of bias. We used the *metabin* and *metacont* functions from the meta package in R 3.6.3 (www.r-project.org) for all meta-analyses. A two-tailed *p* < 0.05 was considered statistically significant.

## Results

### Study Selection

**Figure 1:**
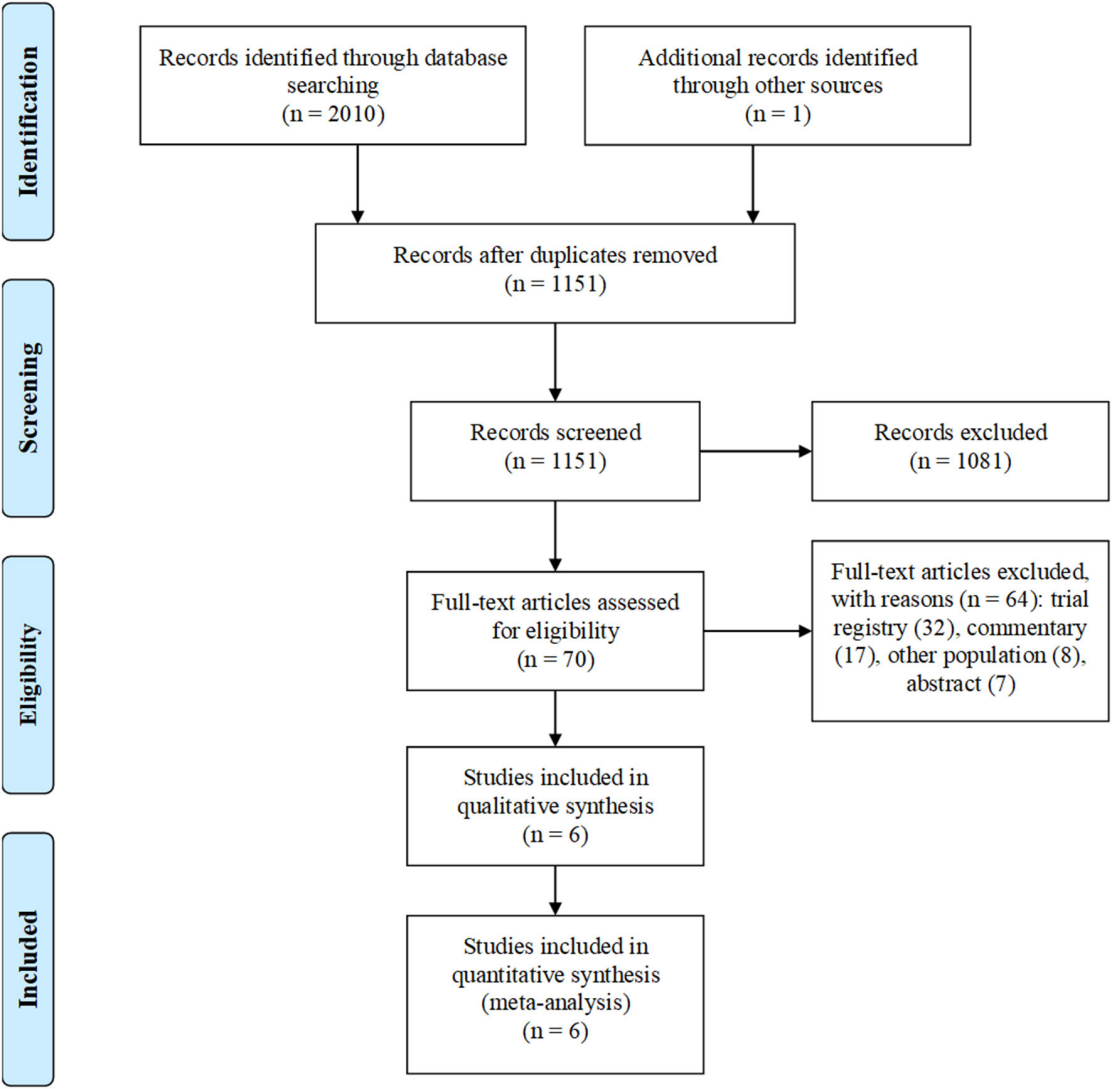
Flow diagram of study selection.

Our search strategy identified initially 2,011 articles. After the removal of duplicates, 1,151 unique articles remained. After screening of studies by title and abstract, 1,081 articles were excluded. After a full-text assessment of 70 articles, 64 articles were excluded: 32 trial registries, 17 commentaries, 8 other populations, and 7 abstracts. Six RCTs were selected for analyses (Figure 1).

### Study Characteristics

Main characteristics of the six included RCTs (*n* = 6,005) are summarized in Table 1. Seventy-nine percent of patients were men, and the mean age ranged between 55.4 and 61 years. The proportion of patients with hypertension (46%) and diabetes (20%) was similar across all trials. Previous MI (15%) was also similar in three trials (20, 21, 23). The sample size (32–4,745 patients) and the length of follow-up (5 days−22.6 months) were heterogeneous across studies. Four trials (20–23) included patients with non–ST-segment elevation MI (NSTEMI) and ST-segment elevation MI (STEMI), whereas in the other two trials (18, 19), only STEMI patients were enrolled. Four RCTs were double-blinded (19–21, 23), one RCT was open-label (18), and in one, RCT blinding was not specified (22).

**Table 1:** Main characteristics of the included studies.

**References**	**Country**	**Type of RCT**	**Population**	**Colchicine**	**Initiation of colchicine**	**Control**	**Time of follow-up**	**Arm**	**Sample size**	**Age (years)**	**Male (%)**	**Discontinuation (%)**	**CV mortality**	**Recurrent MI**	**Follow-up hs-CRP (mg/L)**
Akodad et al. (18)	France	Open-label	STEMI patients with ≤ 12 h successfully treated with PCI	1 mg QD for 30 days	Within first day of acute MI	Standard treatment	30 days	Colchicine	23	60.1 ± 13.1^*^	82%	13%	0/23 (0%)	0/23 (0%)	29 ± 25.6^*^
								Control	22	59.7 ± 11.4^*^	76%	NR	0/21 (0%)	1/21 (4.76%)	21.9 ± 25.4^*^
Deftereos et al. (19)	Greece	Double-blinded	STEMI patients with ≤ 12 h from the onset of chest pain	2 mg loading dose, then 0.5 mg BID for 5 days	After coronary angiography	Placebo	5 days	Colchicine	77	58 (52–54)^†^	70%	26%	NR	NR	42.9 (16.3–71.4)^†^
								Control	74	58 (51–68)^†^	68%	4%	NR	NR	63.8 (34.7–103.4)^†^
Hennessy et al. (20)	Australia	Double-blinded	Patients with type 1 MI within the prior 7 days	0.5 mg BID for 30 days	Within 7 days of acute MI	Placebo	30 days	Colchicine	119	61 ± 13.6^*^	75%	2%	0/119 (0%)	0/119 (0%)	1.6 (0.7–3.5)^†^
								Control	118	61 ± 12.5^*^	79%	4%	0/118 (0%)	2/118 (1.69%)	2 (0.9–4)^†^
Tardif et al. (21)	12 Countries	Double-blinded	Patients with MI <30 days who had completed any planned PCI	0.5 mg QD for 24 months	Within 30 days of MI (13.4 ± 10.2 days)^*^	Placebo	22.6 months (median)	Colchicine	2,366	60.6 ± 10.7^*^	80%	18.4%	20/2,366 (0.84%)	89/2,366 (3.76%)	1.37 (0.75–2.13)^‡^
								Control	2,379	60.5 ± 10.6^*^	82%	18.7%	24/2,379 (1%)	98/2,379 (4.11%)	1.6 (0.9–2.65)^‡^
Wasyanto et al. (22)	Indonesia	Blinding unspecified	Patients with acute MI	0.5 mg QD for 5 days	Not reported	Placebo	5 days	Colchicine	16	57.9^*^	87%	NR	NR	NR	NR
								Control	16	52.9^*^	87%	NR	NR	NR	NR
Tong et al. (23)	Australia	Double-blinded	Patients presenting with ACS (96.7% STEMI or NSTEMI) with evidence of CAD	0.5 mg BID for the first month, followed by 0.5 mg BID for 11 months	After coronary angiography	Placebo	400 days	Colchicine	396	59.7 ± 10.2^*^	81%	15%	3/396 (0.75%)	7/396 (1.76%)	NR
								Control	399	60 ± 10.4^*^	78%	8%	1/399 (0.25%)	11/399 (2.75%)	NR

*RCT, randomized controlled trial; STEMI, ST-segment elevation myocardial infarction; NSTEMI, non–ST-segment elevation myocardial infarction; MI, myocardial infarction; PCI, percutaneous coronary intervention; QD, once a day; BID, twice a day; ACS, acute coronary syndrome; CAD, coronary artery disease; CV, cardiovascular; GI, gastrointestinal; hs-CRP, high-sensitivity C-reactive protein; GI, gastrointestinal; NR, not reported*.

* *Mean ± standard deviation*.

† *Median (interquartile range)*.

‡ *Geometric mean (interquartile range)*.

Control groups were placebo or standard treatment across trials. Doses and treatment duration of colchicine varied among studies. The doses used were 0.5 (21, 22) and 1 (18–20) mg/day across five trials with a duration that ranged from 5 days to 24 months. One trial combined both doses (Table 1) (23). Overall, discontinuation rate of colchicine across trials ranged from 2 to 26% (Table 1). Our search for ongoing trials identified three registered RCTs that assess the effect of colchicine in post–acute MI patients (Supplementary Table 2).

### Risk-of-Bias Assessment

**Figure 2:**
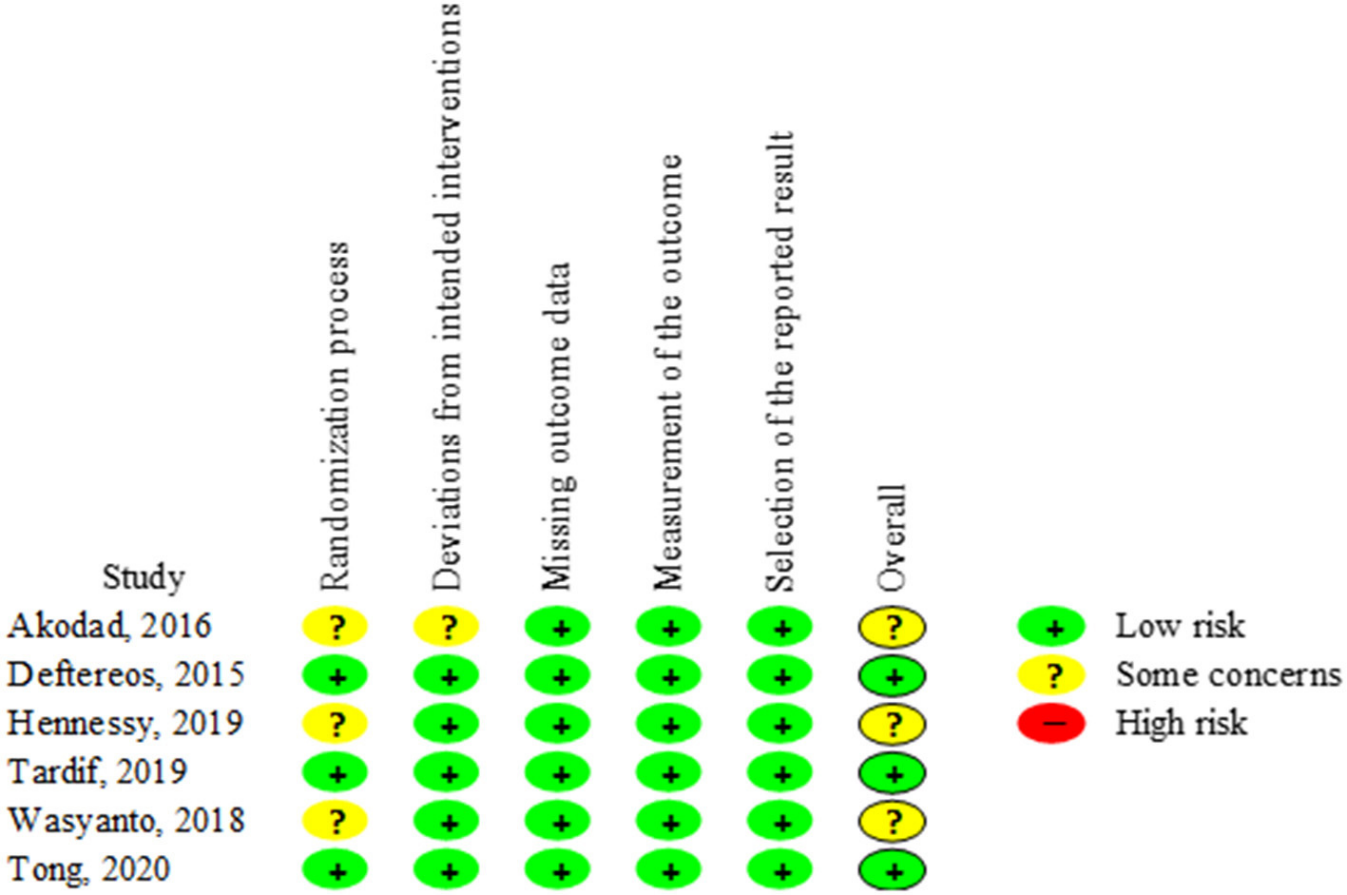
Risk of Bias 2.0 tool for risk-of-bias assessment of included randomized controlled trials.

Three trials demonstrated an overall low risk of bias. The other three trials showed some concerns because they reported insufficient information regarding the randomization process (Figure 2).

### Primary Outcomes

**Figure 3:**
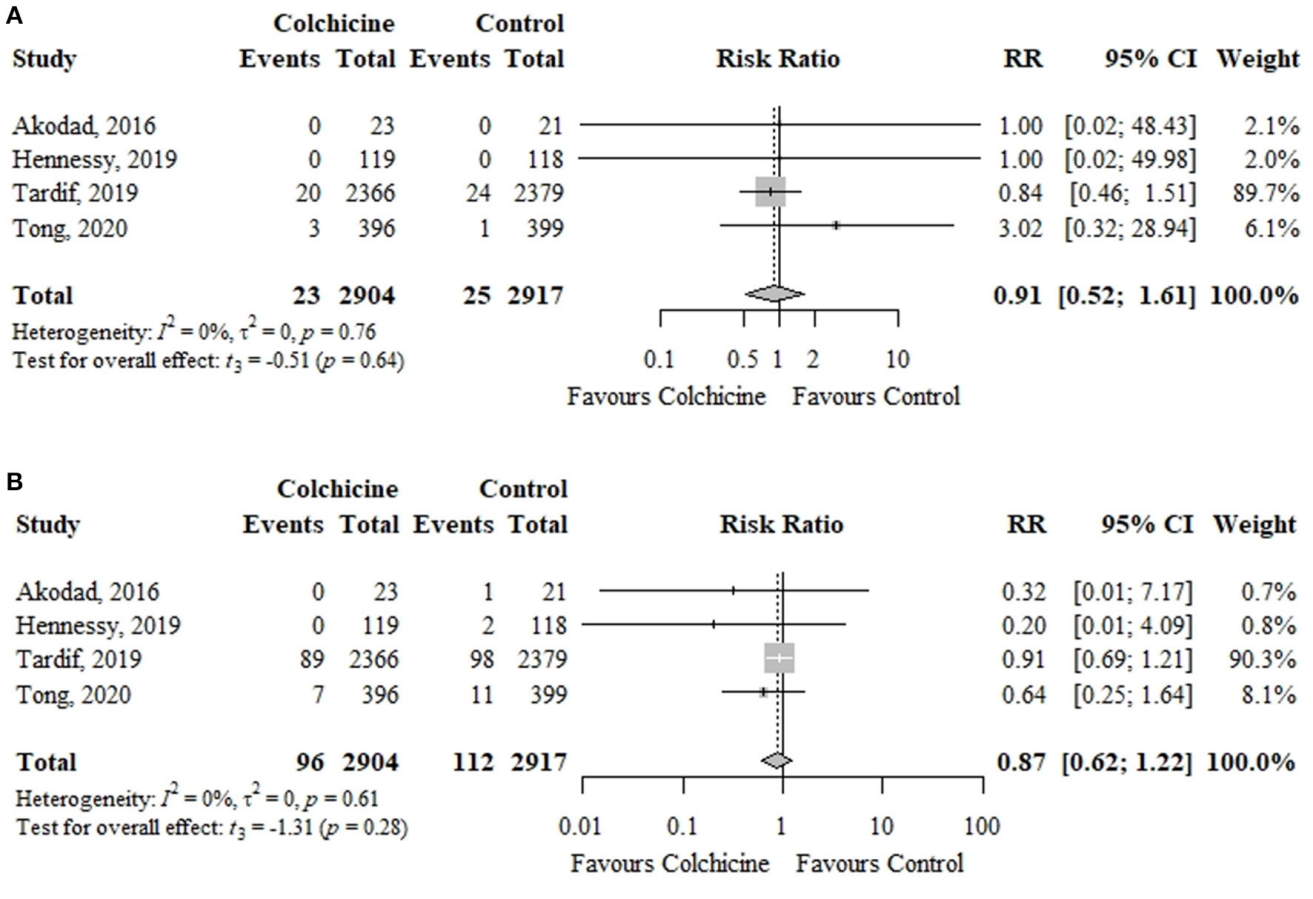
Effect of colchicine vs. control on **(A)** cardiovascular mortality and **(B)** recurrent myocardial infarction. RR, risk ratio; CI, confidence interval.

In four RCTs including 5,821 patients, colchicine did not significantly reduce the risk of cardiovascular mortality (0.79 vs. 0.86%; RR, 0.91; 95% CI, 0.52–1.61; *p* = 0.64; *I*^2^ = 0%) or recurrent MI (3.31 vs. 3.84%; RR, 0.87; 95% CI, 0.62–1.22; *p* = 0.28; *I*^2^ = 0%) in comparison to control group (Figure 3).

### Secondary Outcomes

**Figure 4:**
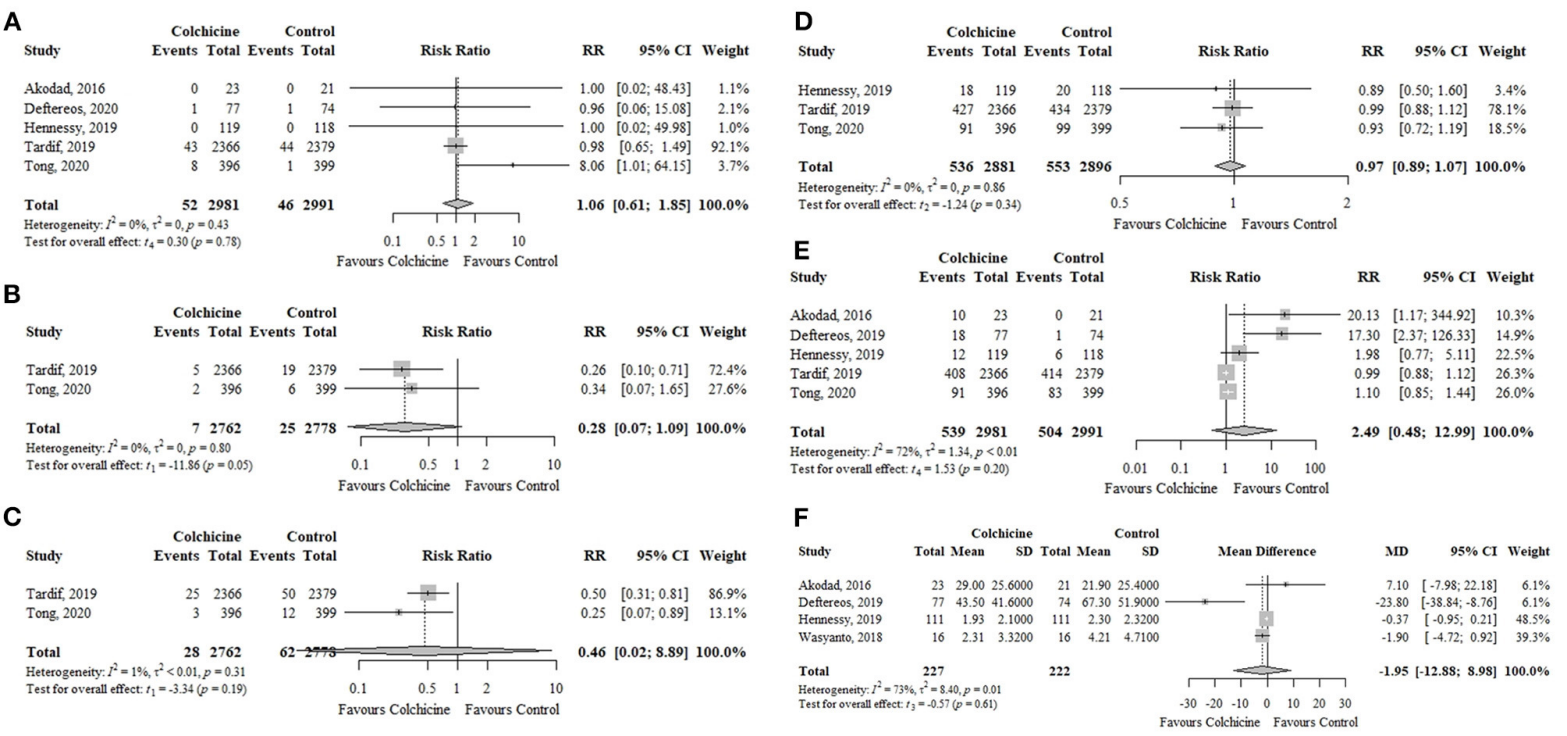
Effects of colchicine on **(A)** all-cause mortality, **(B)** stroke, **(C)** urgent coronary revascularization, **(D)** any adverse events, **(E)** gastrointestinal adverse events, and **(F)** follow-up levels of hs-CRP (mg/L). hs-CRP, high-sensitivity C-reactive protein; RR, risk ratio; CI, confidence interval; SD, standard deviation; MD, mean difference.

Colchicine did not significantly reduce the risk of all-cause mortality (five RCTs; *n* = 5,972; 1.74 vs. 1.54%; RR, 1.06; 95% CI, 0.61–1.85; *p* = 0.78; *I*^2^ = 0%), stroke (two RCTs; *n* = 5,540; 0.25 vs. 0.90%; RR, 0.28; 95% CI, 0.07–1.09; *p* = 0.05; *I*^2^ = 0%), urgent coronary revascularization (two RCTs; *n* = 5,540; 1.01 vs. 2.23%; RR, 0.46; 95% CI, 0.02–8.89; *p* = 0.19; *I*^2^ = 1%), or decreased levels of follow-up hs-CRP (four RCTs; *n* = 449; MD, −1.95 mg/L; 95% CI, −12.88 to 8.98; *p* = 0.61; *I*^2^ = 73%) compared to control group (Figure 4). When assessing sources of high heterogeneity of follow-up hs-CRP levels, the exclusion of the trial by Deftereos et al. (19) found that the effect of colchicine on follow-up hs-CRP levels was similar to the overall analysis and the heterogeneity was reduced (Supplementary Figure 1).

Colchicine did not increase the risk of any adverse events (three RCTs; *n* = 5,777; 18.60 vs. 19.09%; RR, 0.97; 95% CI, 0.89–1.07; *p* = 0.34; *I*^2^ = 0%) or gastrointestinal adverse events (five RCTs; *n* = 5,972; 18.08 vs. 16.85%; RR, 2.49; 95% CI, 0.48–12.99; *p* = 0.20; *I*^2^ = 72%) in comparison to the control group (Figure 4). The most frequently reported gastrointestinal adverse events were nausea, vomiting, and diarrhea. The three trials (18, 19, 23) that provided information on myelotoxicity found no cases in both groups.

### Subgroup Analyses

Subgroup analyses by colchicine dose (0.5 vs. 1 mg/day), time of follow-up (<1 vs. ≥1 year), and treatment duration (≤30 vs. >30 days) did not show differences with the overall analyses for cardiovascular mortality, recurrent MI, all-cause mortality, follow-up levels of hs-CRP, any adverse events, and gastrointestinal adverse events (Supplementary Tables 3–5). The only exceptions were significantly lower risk of urgent coronary revascularization in the trials of Tardif et al. (21) (0.5 mg/day) and Tong et al. (23) (1 mg/day) and a significant risk reduction of stroke with a dose of 0.5 mg/day reported in the trial by Tardif et al. (21) (Supplementary Table 3).

### Sensitivity Analyses

The results of the sensitivity analyses showed that when the meta-analyses were performed without the Hartung–Knapp adjustment, colchicine reduced only the risk of stroke or urgent coronary revascularization in comparison to the control group (Supplementary Table 6). In addition, when only trials with a low risk of bias were pooled, the results were similar to the overall analysis (Supplementary Table 7) except for Deftereos et al. (19) that reported significantly lower levels of hs-CRP in patients treated with colchicine compared to the control group.

### GRADE Summary of Findings

Among the primary outcomes, cardiovascular mortality and recurrent MI had low quality of evidence. There was also a low quality of evidence for all-cause mortality and urgent coronary revascularization. Stroke and any adverse events had a moderate quality of evidence. Finally, gastrointestinal adverse events and follow-up hs-CRP levels were scored as very low quality of evidence (Table 2).

**Table 2:** GRADE summary of findings.

**Outcomes**	Anticipated absolute effects^*^ (95% CI)		**Relative effect (95% CI)**	**No. of participants (studies)**	**Certainty of the evidence (GRADE)**
	**Risk with control**	**Risk with colchicine**			
Cardiovascular mortality follow up: range, 30 days−22.6 months	9 per 1,000	8 per 1,000 (4–14)	RR, 0.91 (0.52–1.61)	5,821 (4 RCTs)	⊕⊕○○ LOW^a^^,^ ^b^
Recurrent myocardial infarction follow up: range, 30 days−22.6 months	38 per 1,000	33 per 1,000 (24–47)	RR, 0.87 (0.62–1.22)	5,821 (4 RCTs)	⊕⊕○○ LOW^a^^,^ ^b^
All-cause mortality follow up: range, 5 days−22.6 months	15 per 1,000	16 per 1,000 (9–28)	RR, 1.06 (0.61–1.85)	5,972 (5 RCTs)	⊕⊕○○ LOW^b^^,^ ^c^
Stroke follow up: range, 400 days−22.6 months	9 per 1,000	3 per 1,000 (1–10)	RR, 0.28 (0.07–1.09)	5,540 (2 RCTs)	⊕⊕⊕○ MODERATE^b^
Urgent coronary revascularization follow up: range, 400 days−22.6 months	22 per 1,000	10 per 1,000 (0–198)	RR, 0.46 (0.02–8.89)	5,540 (2 RCTs)	⊕⊕○○ LOW^b^
High-sensitivity C-reactive protein follow up: range, 5–30 days	—	MD 1.95 mg/L lower (12.88 lower to 8.98 higher)	—	449 (4 RCTs)	⊕○○○ VERY LOW^b^^,^ ^d^^,^ ^e^
Any adverse events follow up: range, 30 days−22.6 months	191 per 1,000	185 per 1,000 (170–204)	RR, 0.97 (0.89–1.07)	5,777 (3 RCTs)	⊕⊕⊕○ MODERATE^f^
Gastrointestinal adverse events follow up: range, 5 days−22.6 months	169 per 1,000	420 per 1,000 (81–1,000)	RR, 2.49 (0.48–12.99)	5,972 (5 RCTs)	⊕○○○ VERY LOW^b^^,^ ^c^^,^ ^g^

* *The risk in the intervention group (and its 95% confidence interval) is based on the assumed risk in the comparison group and the relative effect of the intervention (and its 95% CI). CI, confidence interval; RR, risk ratio; MD, mean difference. The combination of symbols ⊕ and ○ means the degree of certainty of the evidence as follows: very low (⊕○○○), low (⊕⊕○○), moderate (⊕⊕⊕○), and high (⊕⊕⊕⊕)*.

*GRADE Working Group grades of evidence:*

*High certainty: We are very confident that the true effect lies close to that of the estimate of the effect*.

*Moderate certainty: We are moderately confident in the effect estimate: The true effect is likely to be close to the estimate of the effect, but there is a possibility that it is substantially different*.

*Low certainty: Our confidence in the effect estimate is limited: The true effect may be substantially different from the estimate of the effect*.

*Very low certainty: We have very little confidence in the effect estimate: The true effect is likely to be substantially different from the estimate of effect*.

a *Two of four studies had some concerns as risk of bias*.

b *Wide confidence intervals*.

c *Two of five studies had some concerns as risk of bias*.

d *Three of four studies had some concerns as risk of bias*.

e *I^2^ = 73%*.

f *One of three studies had some concerns as risk of bias*.

g *Outcome definition was heterogeneous across studies*.

## Discussion

In our meta-analysis of six RCTs, we found that colchicine did not significantly reduce the risk of cardiovascular mortality, recurrent MI, all-cause mortality, stroke, urgent coronary revascularization, or follow-up levels of hs-CRP compared to placebo or standard treatment. Also, there was no increase in any adverse events or gastrointestinal adverse events associated with the use of colchicine. Overall, both the subgroup analyses by colchicine dose, time of follow-up, or treatment duration and the sensitivity analyses were consistent with the main results.

Colchicine has gained recent attention as a promising anti-inflammatory therapy for patients with CAD. Over the last years, several trials evaluating the role of colchicine as an additional treatment to standard medical therapy for secondary prevention in patients with chronic CAD or after ACS have been published. Colchicine acts by blocking the polymerization of microtubules affecting several functions of inflammatory cells (chemotaxis, adhesion, phagocytosis, and protein excretion), interferes the neutrophil-endothelial interaction through inhibition of the E-selectin expression on endothelial cells, and blocks the activity of the Nod-like protein receptor 3 inflammasome within monocytes and neutrophils at different levels (24–26). In patients with ACS, colchicine acutely suppressed the caspase-1 activity and the local production of proinflammatory interleukins (IL-6, IL-1β, and IL-18) and chemokines, which are key mediators in the inflammatory pathway of the coronary atherothrombosis (27–29). Given the diffuse coronary vascular inflammation observed in patients with acute MI (30), the reduction of the proinflammatory stimulus could lead to the stabilization of both culprit and non-culprit atherosclerotic plaques, decreasing the recurrence of future coronary events. Interestingly, a study in 80 patients with recent ACS found that 1-year treatment with low-dose colchicine favorably modified coronary plaques assessed by coronary computed tomography angiography in comparison with standard medical therapy, irrespective of the reduction of low-density lipoprotein levels (31). In addition, colchicine also decreased monocyte– and neutrophil–platelet aggregation and expression of surface markers of platelet activity, thus targeting the platelet-inflammatory axis (32). On the other hand, preclinical studies based on animal models of acute MI showed that the administration of colchicine produced a reduction of infarct size and cardiac remodeling in the acute and chronic phase post–myocardial injury, probably as a result of the inhibition of the inflammatory response (33–35). These findings were corroborated in a clinical trial of STEMI patients treated with a percutaneous coronary intervention (PCI) that showed a lower infarct size defined by cardiac magnetic resonance in the group allocated to receive high-dose colchicine compared with placebo (19). Overall, these data suggested that colchicine may have a beneficial effect in the setting of remodeling after an acute MI.

Clinical evidence of the impact of colchicine on major adverse cardiovascular outcomes has produced divergent results. The COLCOT trial published by Tardif et al. (21) enrolled the largest population and showed a significant reduction of the primary composite endpoint [hazard ratio (HR), 0.77; 95% CI, 0.61–0.96; *p* = 0.02] in patients with recent MI treated with colchicine compared to placebo. However, this finding was mainly driven by a decrease in stroke and urgent angina revascularization with no effect on cardiovascular mortality (HR, 0.84; 95% CI, 0.46–1.52) or recurrent MI (HR, 0.91; 95% CI, 0.68–1.21). Given that hs-CRP values were measured in only 4.4% of included patients, we decided not to include the hs-CRP values from this RCT (21) in our meta-analysis. Recently, a subanalysis of the COLCOT trial showed that the beneficial effect of colchicine was restricted to patients who started this drug within 3 days post-MI (HR, 0.52; 95% CI, 0.32–0.84), suggesting a possible benefit from early initiation of colchicine when inflammation is more intense (36). In our review, the timing of colchicine initiation in three RCTs (18, 19, 23) was in the acute phase of MI. In contrast, in the LoDoCo-MI (20) and COLCOT trials, the initiation was within 7 and 30 days (mean of 13.4 ± 10.2 days) after the index event, respectively. Because of the lack of accurate reporting of treatment onset, a subgroup analysis was not possible. The COPS trial published by Tong et al. (23) showed that colchicine did not significantly decrease cardiovascular outcomes at 400 days in patients with ACS. In contrast, an increased risk of all-cause mortality (HR, 8.20; 95% CI, 1.03–65.61) was reported in the colchicine group compared to the control group. The COLCOT and COPS trials were the only ones to report the risk of stroke and urgent coronary revascularization. Although both RCTs reported individual favorable results for these outcomes, the pooled effect estimates using the Hartung–Knapp adjustment were not significant. However, additional caution with the interpretation of CIs is required when there are RCTs of very unequal sizes as in our review (15). Shah et al. (37) reported that a single dose of oral pre-procedural colchicine did not reduce target vessel revascularization myocardial injury or 30-day major adverse cardiovascular events, but it did reduce post-procedure hs-CRP levels. This study was excluded from our review because the population was composed of patients with CAD without adequate stratified randomization for the subgroup with acute MI. Importantly, the treatment duration of colchicine was heterogeneous across trials. In four of six RCTs (18, 20, 21, 23), the duration was 30 days or shorter, whereas in the remaining two RCTs (19, 22), it was 12 months or longer. The inhomogeneity in the initiation and duration of colchicine in our review raises the need that adequately powered RCTs with earlier and prolonged treatment are required. Currently, there is one large trial (38), which is recruiting 7,000 patients with MI who have undergone PCI. The CLEAR SINERGY (Colchicine and Spironolactone in Patients with MI/SYNERGY Stent Registry) trial has a 2 × 2 factorial design where patients are randomized to colchicine vs. placebo and spironolactone vs. placebo with a primary outcome defined as the composite of death, recurrent target vessel MI, stroke, or ischemia-driven target vessel revascularization (38).

It has been largely recognized that hs-CRP is an inflammatory biomarker with important prognostic value in the setting of acute MI (39). High levels of hs-CRP following an acute coronary event have been associated with increased short- and long-term risk of recurrent cardiovascular events and mortality (39, 40). There are conflicting data on whether colchicine can reduce hs-CRP levels in patients with CAD (5, 8). Our study found no significant difference on pooled hs-CRP levels during follow-up between patients treated with colchicine or placebo. Only Deftereos et al. (19) reported a significant reduction of hs-CRP levels in the colchicine group. This finding may be related to the administration of a loading dose of colchicine during primary PCI.

Historically, colchicine has been considered a drug with an acceptable safety profile. The most common reported adverse effects are gastrointestinal (diarrhea, nausea, and vomiting), which may occur in 5–10% of patients (41). Rarely can colchicine cause myopathy, rhabdomyolysis, and myelosuppression (41). Overall, our review shows the safety of colchicine, with an incidence of nearly 18% of gastrointestinal adverse events in the active group but without a pooled significant difference when colchicine was compared with the control group (placebo or standard treatment). This finding was consistent across RCTs, except for Akodad et al. (18) and Deftereos et al. (19) that reported more gastrointestinal adverse events in patients treated with colchicine, which is probably related to the use of higher doses of colchicine. Moreover, Tardif et al. (21) found an increase in pneumonia cases in the colchicine group in comparison to placebo (0.9 vs. 0.4%).

There are several previously published systematic reviews examining the effect of colchicine in patients with CAD (Supplementary Table 8) (5–11). Two reviews (5, 7) concluded that colchicine was not associated with a significant reduction of major adverse cardiovascular events or death in patients with CAD. The other five reviews (6, 8–11) concluded that colchicine reduces the incidence of future cardiovascular events. Our systematic review included RCTs that assessed colchicine in post–acute MI patients and performed further subgroup analyses according to colchicine dose, time of follow-up, and treatment duration. Conversely, prior reviews did not specify if all CAD patients suffered from acute MI. One review included patients with ACS; however, only a narrative synthesis was conducted (8). Moreover, six reviews (5–7, 9–11) pooled data from patients with chronic coronary syndrome and ACS, despite the fact that these populations are clinically different. In contrast to our findings, four previous reviews (7–9, 11) reported that colchicine was associated with an increased risk of gastrointestinal adverse events. Only one review used the Hartung–Knapp adjustment for CIs (5). Besides, quality of evidence per outcome using GRADE methodology was evaluated in only one review (5). In contrast to prior reviews, we intended to assess the efficacy and safety in a high risk CAD population (post–acute MI); therefore, the usefulness of our results is more applicable to daily clinical practice.

Our study has some limitations. First, the number of pooled RCTs is low for all outcomes. However, we adjusted all 95% CIs using the Hartung–Knapp method to address possible type I error with the conventional random-effects approach. Second, the population of included RCTs was heterogeneous in terms of the type of patients (NSTEMI and STEMI), the dosage of colchicine, and the timing of initiation. Third, given that RCTs reported composite primary outcomes with heterogeneous definitions, they were not included in our review. Fourth, the treatment duration and follow-up for most of the included RCTs were relatively short (≤30 days). Therefore, it is not possible to know if treatment with colchicine for a longer period of time may be beneficial. Although we conducted a subgroup analyses according to the duration of treatment (≤30 vs. >30 days) and follow-up duration (<1 vs. ≥1 year), which showed no differences between both groups, these results should be considered with caution due to the low number of trials and the secondary nature of the analyses. Finally, the dose and duration of colchicine were inconsistent across groups. Although subgroup analyses revealed that no dose (0.5 vs. 1 mg/day) and duration (≤30 vs. >30 days) of colchicine reduced cardiovascular outcomes, these results should be considered only exploratory.

## Conclusion

Our meta-analysis suggests that compared with placebo or standard treatment, colchicine does not reduce cardiovascular mortality, recurrent MI, all-cause mortality, stroke, urgent coronary revascularization, and levels of follow-up hs-CRP. In addition, colchicine does not increase the risk of adverse events, including gastrointestinal events. However, more RCTs with larger sample sizes are needed to assess the long-term benefits or harms of colchicine.

## Data Availability Statement

The original contributions presented in the study are included in the article/Supplementary Material, further inquiries can be directed to the corresponding author/s.

## Author Contributions

All authors listed have made a substantial, direct and intellectual contribution to the work, and approved it for publication.

## Conflict of Interest

VP was employed by the company MedErgy HealthGroup, Inc. The remaining authors declare that the research was conducted in the absence of any commercial or financial relationships that could be construed as a potential conflict of interest.
