# Supplementary material for: Efficacy and Safety of Colchicine in Post–acute Myocardial Infarction Patients: A Systematic Review and Meta-Analysis of Randomized Controlled Trials
Source: Front Cardiovasc Med. 2021 Jun 8;8:676771. doi: 10.3389/fcvm.2021.676771 (PMC8217746; doi:10.3389/fcvm.2021.676771)
Supplement: Supplementary file 1 [file Data_Sheet_1.doc]

**SUPPLEMENTARY DATA**

**Efficacy and safety of colchicine in post-acute myocardial infarction patients: a systematic review and meta-analysis of randomized controlled trials**

Diaz-Arocutipa C et al.

**Supplementary Table 1**. Electronic search strategy

| **PubMed**  (colchicine[mh] OR colchicine[tiab]) AND (“myocardial infarction”[mh] OR “myocardial infarction”[tiab] OR “coronary artery disease”[mesh] OR “coronary artery disease”[tiab] OR “acute coronary syndrome”[mh] OR “acute coronary syndrome”[tiab] OR “angina pectoris”[mh] OR “angina”[tiab] OR “ischemic heart disease”[tiab] OR “coronary heart disease”[tiab] OR “heart attack”[tiab] OR “myocardial ischemia”[mh] OR “coronary disease”[mh] OR “coronary thrombosis”[mh] OR “coronary stenosis”[mh] OR “coronary occlusion”[mh]) AND ((“randomized controlled trial”[pt] OR “controlled clinical trial”[pt] OR randomized[tiab] OR placebo[tiab] OR “drug therapy”[sh] OR randomly[tiab] OR trial[tiab] OR groups[tiab]) NOT (animals[mh] NOT humans[mh])) |
| --- |
| **Embase**  (colchicine/exp OR colchicine) AND (‘myocardial infarction’/exp OR ‘myocardial infarction’ OR ‘coronary artery disease’/exp OR ‘coronary artery disease’ OR ‘acute coronary syndrome’/exp OR ‘acute coronary syndrome’ OR ‘angina pectoris’/exp OR ‘angina pectoris’ OR angina/exp OR angina OR ‘ischemic heart disease’/exp OR ‘ischemic heart disease’ OR ‘coronary heart disease’/exp OR ‘coronary heart disease’ OR ‘heart attack’/exp OR ‘heart attack’ OR ‘myocardial ischemia’/exp OR ‘myocardial ischemia’ OR ‘coronary disease’/exp OR ‘coronary disease’ OR ‘coronary thrombosis’/exp OR ‘coronary thrombosis’ OR ‘coronary stenosis’/exp OR ‘coronary stenosis’ OR ‘coronary occlusion’/exp OR ‘coronary occlusion’) |
| **Scopus**  TITLE-ABS-KEY (colchicine AND (“myocardial infarction” OR “coronary artery disease” OR “acute coronary syndrome” OR angina OR “ischemic heart disease” OR “coronary heart disease” OR “heart attack” OR “myocardial ischemia” OR “coronary disease” OR “coronary thrombosis” OR “coronary stenosis” OR “coronary occlusion”)) |
| **Web of Science**  All=(colchicine AND (“myocardial infarction” OR “coronary artery disease” OR “acute coronary syndrome” OR angina OR “ischemic heart disease” OR “coronary heart disease” OR “heart attack” OR “myocardial ischemia” OR “coronary disease” OR “coronary thrombosis” OR “coronary stenosis” OR “coronary occlusion”)) |
| **CENTRAL**  (colchicine AND (“myocardial infarction” OR “coronary artery disease” OR “acute coronary syndrome” OR angina OR “ischemic heart disease” OR “coronary heart disease” OR “heart attack” OR “myocardial ischemia” OR “coronary disease” OR “coronary thrombosis” OR “coronary stenosis” OR “coronary occlusion”)):ti,ab,kw |

**Supplementary Table 2**. Ongoing randomized controlled trials (as of October 20, 2020)

| Trial ID | Trial title | Experimental arm | Control arm | Country | Estimated enrollment | Estimated study completion date |
| --- | --- | --- | --- | --- | --- | --- |
| NCT04218786 | Effect of Colchicine in Patients With Myocardial Infarction | Colchicine 0.5 mg/day for three months | Placebo | Pakistan | 800 patients | November 2020 |
| NCT04420624 | Colchicine to Prevent Sympathetic Denervation After an Acute Myocardial Infarction (COLD-MI) | Colchicine 0.5 or 1 mg/day for one month | Standard treatment | France | 56 patients | September 2021 |
| NCT03156816 | Colchicine for Left Ventricular Remodeling Treatment in Acute Myocardial Infarction (COVERT-MI) | Colchicine 2mg bolus, then 1 mg/day for five days | Placebo | France | 194 patients | January 2022 |
| NCT03048825 | Colchicine and Spironolactone in Patients With MI / SYNERGY Stent Registry (CLEAR SYNERGY) | Colchicine 0.5 mg/day, spironolactone 25 mg/day | Placebo | Canada | 7000 patients | March, 2025 |

.

**Supplementary Table 3**. Subgroup analyses according to colchicine dose

| Outcomes | Number of studies | Effect measures | 95%CI | p-value | I^2^ |
| --- | --- | --- | --- | --- | --- |
| Cardiovascular mortality |  |  |  |  |  |
| Colchicine 0.5 mg/day | 2 | RR: 0.84 | 0.60-1.17 | 0.10 | 0% |
| Colchicine 1 mg/day | 2 | RR: 2.28 | 0.01-1030.06 | 0.34 | 0% |
| Recurrent myocardial infarction |  |  |  |  |  |
| Colchicine 0.5 mg/day | 2 | RR: 0.90 | 0.15-5.37 | 0.59 | 0% |
| Colchicine 1 mg/day | 2 | RR: 0.61 | 0.05-6.76 | 0.23 | 0% |
| All-cause mortality |  |  |  |  |  |
| Colchicine 0.5 mg/day | 2 | RR: 0.98 | 0.96-1.01 | 0.07 | 0% |
| Colchicine 1 mg/day | 3 | RR: 3.05 | 0.12-75.12 | 0.27 | 0% |
| Urgent coronary revascularization |  |  |  |  |  |
| Colchicine 0.5 mg/day | 1 | RR: 0.50 | 0.31-0.81 | <0.01 | - |
| Colchicine 1 mg/day | 1 | RR: 0.25 | 0.07-0.89 | 0.03 | - |
| Stroke |  |  |  |  |  |
| Colchicine 0.5 mg/day | 1 | RR: 0.26 | 0.10-0.71 | <0.01 | - |
| Colchicine 1 mg/day | 1 | RR: 0.34 | 0.07-1.65 | 0.18 | - |
| Follow-up levels of hs-CRP |  |  |  |  |  |
| Colchicine 0.5 mg/day | 2 | MD: -0.49 | -5.62 to 4.65 | 0.44 | 8% |
| Colchicine 1 mg/day | 2 | MD: -8.36 | -204.67 to 187.96 | 0.68 | 88% |
| Any adverse events |  |  |  |  |  |
| Colchicine 0.5 mg/day | 2 | RR: 0.99 | 0.76-1.28 | 0.60 | 0% |
| Colchicine 1 mg/day | 1 | RR: 0.93 | 0.72-1.19 | 0.54 | - |
| Gastrointestinal adverse events |  |  |  |  |  |
| Colchicine 0.5 mg/day | 2 | RR: 1.19 | 0.02-57.37 | 0.67 | 51% |
| Colchicine 1 mg/day | 3 | RR: 5.10 | 0.07-365.13 | 0.24 | 82% |
| hs-CRP, high-sensitivity C-reactive protein; 95%CI, 95% confidence interval; RR, risk ratio; MD, mean difference. | | | | | |

**Supplementary Table 4. Subgroup analyses according** to time of follow-up

| Outcomes | Number of studies | Effect measures | 95%CI | p-value | I^2^ |
| --- | --- | --- | --- | --- | --- |
| Cardiovascular mortality |  |  |  |  |  |
| Follow-up <1 year | 2 | RR: 1 | 1-1 | 0.21 | 0% |
| Follow-up ≥1 year | 2 | RR: 0.98 | 0-212.12 | 0.97 | 14% |
| Recurrent myocardial infarction |  |  |  |  |  |
| Follow-up <1 year | 2 | RR: 0.25 | 0.01-5.42 | 0.11 | 0% |
| Follow-up ≥1 year | 2 | RR: 0.89 | 0.26-3.05 | 0.43 | 0% |
| All-cause mortality |  |  |  |  |  |
| Follow-up <1 year | 3 | RR: 0.98 | 0.92-1.04 | 0.29 | 0% |
| Follow-up ≥1 year | 2 | RR: 2.18 | 0-936380.9 | 0.58 | 74% |
| Urgent coronary revascularization |  |  |  |  |  |
| Follow-up <1 year | 0 | - | - | - | - |
| Follow-up ≥1 year | 2 | RR: 0.46 | 0.02-8.89 | 0.19 | 1% |
| Stroke |  |  |  |  |  |
| Follow-up <1 year | 0 | - | - | - | - |
| Follow-up ≥1 year | 2 | RR: 0.28 | 0.07-1.09 | 0.05 | 0% |
| Follow-up levels of hs-CRP |  |  |  |  |  |
| Follow-up <1 year | 4 | MD: -1.95 | -12.88 to 8.98 | 0.61 | 73% |
| Follow-up ≥1 year | 0 | - | - | - | - |
| Any adverse events |  |  |  |  |  |
| Follow-up <1 year | 1 | RR: 0.89 | 0.50-1.60 | 0.70 | - |
| Follow-up ≥1 year | 2 | RR: 0.98 | 0.70-1.36 | 0.53 | 0% |
| Gastrointestinal adverse events |  |  |  |  |  |
| Follow-up <1 year | 3 | RR: 6.07 | 0.20-180.16 | 0.15 | 62% |
| Follow-up ≥1 year | 2 | RR: 1.01 | 0.59-1.72 | 0.84 | 0% |
| hs-CRP, high-sensitivity C-reactive protein; 95%CI, 95% confidence interval; RR, risk ratio; MD, mean difference. | | | | | |

**Supplementary Table 5**. Subgroup analyses according to treatment duration

| Outcomes | Number of studies | Effect measures | 95%CI | p-value | I^2^ |
| --- | --- | --- | --- | --- | --- |
| Cardiovascular mortality |  |  |  |  |  |
| ≤30 days | 2 | RR: 1 | 1-1 | 0.21 | 0% |
| >30 days | 2 | RR: 0.98 | 0-212.12 | 0.97 | 14% |
| Recurrent myocardial infarction |  |  |  |  |  |
| ≤30 days | 2 | RR: 0.25 | 0.01-5.42 | 0.11 | 0% |
| >30 days | 2 | RR: 0.89 | 0.26-3.05 | 0.43 | 0% |
| All-cause mortality |  |  |  |  |  |
| ≤30 days | 3 | RR: 0.98 | 0.92-1.04 | 0.29 | 0% |
| >30 days | 2 | RR: 2.18 | 0-936380.9 | 0.58 | 74% |
| Urgent coronary revascularization |  |  |  |  |  |
| ≤30 days | 0 | - | - | - | - |
| >30 days | 2 | RR: 0.46 | 0.02-8.89 | 0.19 | 1% |
| Stroke |  |  |  |  |  |
| ≤30 days | 0 | - | - | - | - |
| >30 days | 2 | RR: 0.28 | 0.07-1.09 | 0.05 | 0% |
| Follow-up levels of hs-CRP |  |  |  |  |  |
| ≤30 days | 4 | MD: -1.95 | -12.88 to 8.98 | 0.61 | 73% |
| >30 days | 0 | - | - | - | - |
| Any adverse events |  |  |  |  |  |
| ≤30 days | 1 | RR: 0.89 | 0.50-1.60 | 0.70 | - |
| >30 days | 2 | RR: 0.98 | 0.70-1.36 | 0.53 | 0% |
| Gastrointestinal adverse events |  |  |  |  |  |
| ≤30 days | 3 | RR: 6.07 | 0.20-180.16 | 0.15 | 62% |
| >30 days | 2 | RR: 1.01 | 0.59-1.72 | 0.84 | 0% |
| hs-CRP, high-sensitivity C-reactive protein; 95%CI, 95% confidence interval; RR, risk ratio; MD, mean difference. | | | | | |

**Supplementary Table 6**. Sensitivity analyses without the Hartung-Knapp adjustment

| Outcomes | Number of studies | Effect measures | 95%CI | p-value | I^2^ |
| --- | --- | --- | --- | --- | --- |
| Cardiovascular mortality | 4 | RR: 0.91 | 0.52-1.60 | 0.75 | 0% |
| Recurrent myocardial infarction | 4 | RR: 0.87 | 0.67-1.14 | 0.31 | 0% |
| All-cause mortality | 5 | RR: 1.06 | 0.71-1.58 | 0.77 | 0% |
| Urgent coronary revascularization | 2 | RR: 0.46 | 0.29-0.73 | <0.01 | 1% |
| Stroke | 2 | RR: 0.28 | 0.12-0.65 | <0.01 | 0% |
| Follow-up levels of hs-CRP | 4 | MD: -1.95 | -5.93 to 2.03 | 0.34 | 73% |
| Any adverse events | 3 | RR: 0.97 | 0.88-1.08 | 0.63 | 0% |
| Gastrointestinal adverse events | 5 | RR: 2.49 | 0.78-7.99 | 0.13 | 72% |
| hs-CRP, high-sensitivity C-reactive protein; 95%CI, 95% confidence interval; RR, risk ratio; MD, mean difference. | | | | | |

**Supplementary Table 7**. Sensitivity analyses including only trials with low risk of bias

| Outcomes | Number of studies | Effect measures | 95%CI | p-value | I^2^ |
| --- | --- | --- | --- | --- | --- |
| Cardiovascular mortality | 2 | RR: 0.98 | 0-212.12 | 0.97 | 14% |
| Recurrent myocardial infarction | 2 | RR: 0.89 | 0.26-3.05 | 0.43 | 0% |
| All-cause mortality | 3 | RR: 1.59 | 0.11-23.62 | 0.54 | 47% |
| Urgent coronary revascularization | 2 | RR: 0.46 | 0.02-8.89 | 0.19 | 1% |
| Stroke | 2 | RR: 0.28 | 0.07-1.09 | 0.05 | 0% |
| Follow-up levels of hs-CRP | 1 | MD: -23.80 | -38.84 to -8.76 | <0.01 | - |
| Any adverse events | 2 | RR: 0.98 | 0.70-1.36 | 0.53 | 0% |
| Gastrointestinal adverse events | 3 | RR: 2.09 | 0.05-83.42 | 0.48 | 76% |
| hs-CRP, high-sensitivity C-reactive protein; 95%CI, 95% confidence interval; RR, risk ratio; MD, mean difference. | | | | | |

**Supplementary Table 8. Comparison of published systematic reviews on the use of colchicine in patients with coronary artery disease**

|  | Our study | Xia et al. | Xiang et al. | Al-Abdouh et al. | McKnight et al. | Samuel et al. | Tien et al. | Ullah et al. |
| --- | --- | --- | --- | --- | --- | --- | --- | --- |
| Year of publication | 2021 | 2021 | 2021 | 2020 | 2020 | 2020 | 2020 | 2020 |
| Aim | To evaluate the efficacy and safety of colchicine in patients post-acute MI | To determine the clinical utility of colchicine treatment in patients with CAD | To evaluate the efficacy and safety of colchicine in the secondary prevention of CAD | To evaluate the benefits of colchicine in patients with CAD including stable and after ACS | To evaluate the efficacy and safety of colchicine for secondary prevention after ACS | To evaluate the efficacy and safety of colchicine for secondary CV prevention among patients with clinically manifest CAD | To evaluate the CV protective effects of colchicine on patients with CAD | To bring consensus on the clinical use of colchicine in patients with stable and non-stable CAD |
| Search databases | PubMed, Embase, Scopus, Web of Science, CENTRAL | PubMed, Cochrane, Scopus | PubMed, Embase, CENTRAL, Web of Science, Google Scholar | PubMed, Embase, Cochrane | Medline, Embase | PubMed, Embase, CENTRAL | PubMed, Embase | Medline, Embase, Cochrane |
| Search cut-off date | January 18, 2021 | August, 2020 | August 31, 2020 | February 28, 2020 | June, 2020 | September 1, 2020 | April 28, 2020 | December 2, 2019 |
| Population | Patients post-acute MI | Patients with CCS and ACS | Patients with CCS and ACS | Patients with CCS and ACS | Patients with ACS | Patients with CCS and ACS | Patients with CCS and ACS | Patients with CCS and ACS |
| Included studies | 6 RCTs | 5 RCTs | 8 RCTs | 6 RCTs | 9 RCTs | 4 RCTs | 10 RCTs | 6 RCTs |
| Total number of patients | 6005 patients | 11790 patients | 11463 patients | 6154 patients | 5756 patients | 11594 patients | 6699 patients | 5820 patients |
| Risk of bias assessment | RoB 2.0 tool | RoB 1.0 tool | RoB 2.0 tool | RoB 1.0 tool | Jadad Scale | RoB 2.0 tool | RoB 1.0 tool | RoB 1.0 tool |
| Outcomes | CV mortality, recurrent MI, all-cause mortality, stroke, urgent coronary revascularization, hs-CRP, any adverse events, and GI adverse events | MACE, CV mortality, MI, urgent revascularization, stroke, non-CV mortality, GI events | MACE, all-cause mortality, urgent coronary revascularization, stroke, acute MI, diarrhea | MACE, myocardial infarction (MI), all-cause mortality, CV mortality, stroke | CRP, cytokines, infarct size, CV events | MACE, CV mortality, MI, stroke, urgent coronary revascularization, deep vein thrombosis or pulmonary embolus, atrial fibrillation, non-CV mortality, infection, pneumonia, hospitalization for GI event, diagnosis of cancer | MI, restenosis after PCI, all-cause mortality, GI events | MACE, mortality, ACS, cardiac arrest, stent restenosis, revascularization, stroke, GI adverse events |
| Conclusion | In patients with MI, colchicine does not reduce CV or all-cause mortality, recurrent MI, or other CV outcomes. Also, colchicine not increase drug-related adverse events | Colchicine treatment may reduce the risk of future CV events in CAD patients | Colchicine is an accessible, safe, and effective drug that could be successfully utilized for the secondary prevention of CAD | Colchicine was not associated with a significant decrease in CV endpoints and mortality in patients with CAD | Adjunctive colchicine 0.5 mg daily for greater than 30 days is reasonable for an ACS population on guideline-directed medical therapy treated with PCI | The addition of low-dose colchicine to standard medical therapy reduces the incidence of major CV events, except CV mortality, when compared to standard medical therapy alone | There is a decreased composite risk of MI and restenosis after PCI with the use of colchicine in patients with CAD. However, colchicine did not appear beneficial for all-cause mortality, and it led to a higher risk of GI events | In patients with CAD presenting with an ACS or stable angina, colchicine might offer no significant reduction in MACE and could potentially be harmful due to a significantly higher risk of GI-related adverse events |

MI, myocardial infarction; CAD, coronary artery disease; CCS, chronic coronary syndrome; ACS, acute coronary syndrome; CV, cardiovascular; CENTRAL, Cochrane Controlled Register of Trials; RCTs, randomized controlled trials; hs-CRP, high-sensitivity C-reactive protein; RoB, risk of bias; MACE, major adverse cardiovascular events; GI, gastrointestinal; PCI, percutaneous coronary intervention.


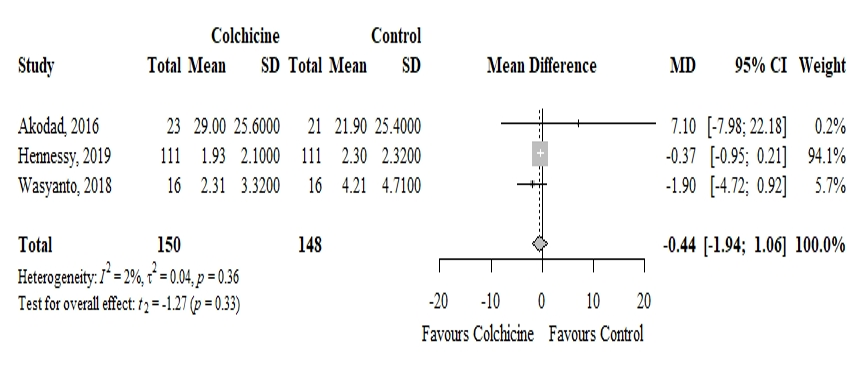


**Supplementary Figure 1. Sensitivity analysis by excluding Deftereos et al. trial of the effect of colchicine on follow-up levels of high-sensitivity C-reactive protein (mg/L). SD indicates standard deviation; MD, mean difference; CI; confidence interval.**
